# A rare case of inflammatory polyp in the common bile duct with cholangitis

**DOI:** 10.1002/deo2.143

**Published:** 2022-07-01

**Authors:** Kazunori Nakaoka, Senju Hashimoto, Naoto Kawabe, Teiji Kuzuya, Hiroyuki Tanaka, Takuji Nakano, Yuichiro Uchida, Yohei Miyachi, Kohei Funasaka, Mitsuo Nagasaka, Yoshihito Nakagawa, Takeshi Takahara, Ryoji Miyahara, Tomoyuki Shibata, Tetsuya Tsukamoto, Koichi Suda, Yoshiki Hirooka

**Affiliations:** ^1^ Department of Gastroenterology and Hepatology Fujita Health University Aichi Japan; ^2^ Department of Surgery Fujita Health University Aichi Japan; ^3^ Department of Diagnostic Pathology Fujita Health University Aichi Japan

**Keywords:** cholangitis, common bile duct stones, endoscopic retrograde cholangiopancreatography, inflammatory polyp, peroral cholangioscopy

## Abstract

The diagnosis of bile duct tumors can be difficult at times. A transpapillary bile duct biopsy findings with endoscopic retrograde cholangiopancreatography sometimes contradict diagnostic imaging findings. In bile duct tumors, inflammatory polyps in the extrahepatic bile duct are relatively rare with extrahepatic cholangitis. The disease's clinical relevance, including its natural history and prognosis, is not always clear. We show here a rare case of an inflammatory polyp in the common bile duct. A 69‐year‐old woman with abdominal pain was diagnosed with cholangitis. The findings of contrast‐enhanced computed tomography and magnetic resonance cholangiopancreatography suggested that she had extrahepatic cholangiocarcinoma. The examination and therapy of cholangitis were performed by endoscopic retrograde cholangiopancreatography. The cholangiography revealed a suspected tumor in the hilar bile duct with some common bile duct stones. Then, after endoscopic sphincterotomy to remove tiny common bile duct stones, further detailed examinations were performed at the same time using an oral cholangioscope revealed a papillary raised lesion with a somewhat white surface in the bile duct; a biopsy was conducted on the same spot, and epithelial cells with mild atypia appeared in the shape of a papilla. Since the malignant tumor or the intraductal papillary neoplasm of the bile duct could not be ruled out, extrahepatic bile duct resection was conducted with the patient's informed consent. Bile duct inflammatory polyp was the histopathological diagnosis.

## INTRODUCTION

The diagnostic imaging findings by cholangioscopy are useful for bile duct diagnosis.^1^ However, precise preoperative diagnosis of a biliary tumor is still challenging. Biopsy and direct observation with a peroral cholangioscopy (POCS) with endoscopic retrograde cholangiopancreatography (ERCP) diagnoses can sometimes contradict diagnostic imaging findings. Malignant tumors and intraductal papillary neoplasm of the bile duct (IPNB) can be used to differentiate papillary bile duct tumors. The clinical entity IPNB was defined in 2010.^2^ IPNB is characterized by papillary growth and encompasses intraductal papillary cholangiocarcinoma and its precursor lesions. The majority of prior cases of common bile duct (CBD) polyps may now be classified as IPNB.^3^ Because IPNB has a high malignant potential and frequently causes recurrent cholangitis and obstructive jaundice, surgical excision is indicated in the absence of distant metastases. On the other hand, inflammatory polyps of CBD are uncommon, and knowledge of their origin and evolution is restricted. We present a case of cholangitis caused by a solitary inflammatory polyp in the CBD.

## CASE REPORT

**FIGURE 1:**
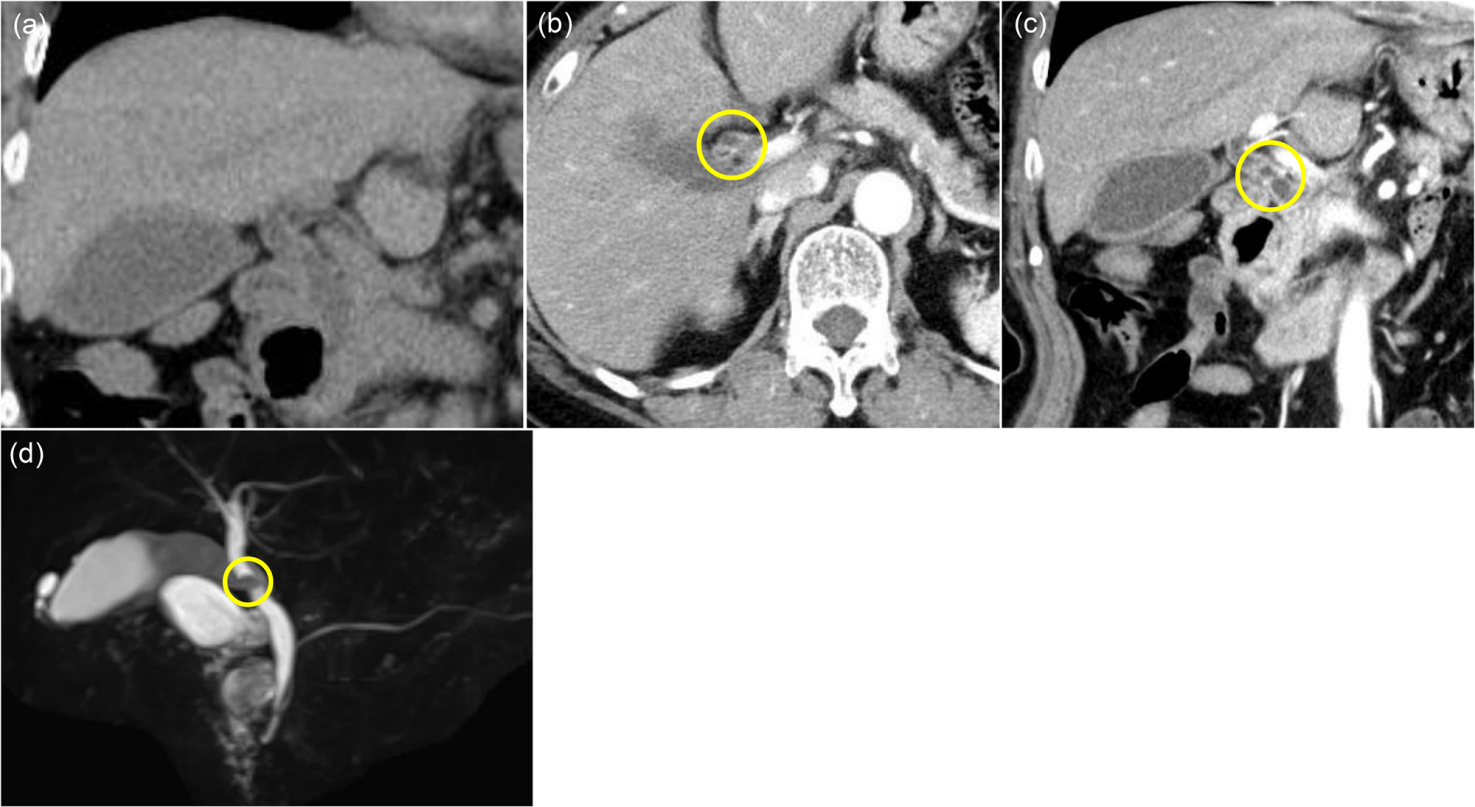
(a) Computed tomography. Common bile duct stones, gallbladder enlargements, and intrahepatic bile duct dilations were all visible and discovered on plain computed tomography. (b,c) Contrast‐enhanced computed tomography revealed thickening of the bile duct wall in the bile duct in the hilar region of the liver, and an enhancing effect is observed at the same location (yellow circle). (d) Magnetic resonance cholangiopancreatography revealed a lack of signal at the junction of the hilar region bile duct and the distal bile duct (yellow circle)

**FIGURE 2:**
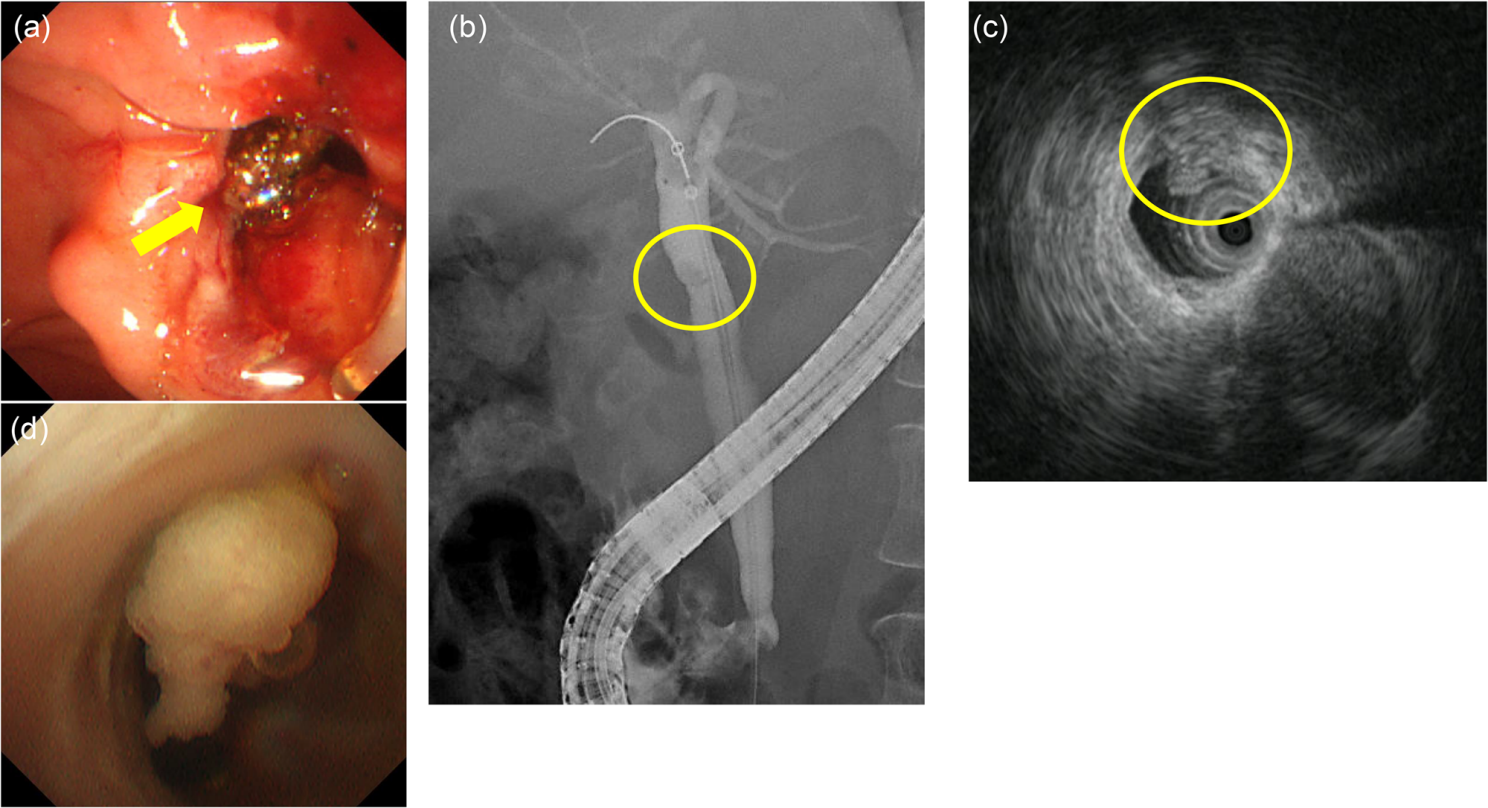
Endoscopic retrograde cholangiopancreatography. (a) To remove small common bile duct stones, an endoscopic sphincterotomy was performed (yellow arrow). (b) The cholangiography showed an immobile translucent image in the hilar bile duct (yellow circle). (c) The findings of intraductal ultrasonography imaging revealed that the tumor has invaded the bile duct epithelium, although the bile duct's exterior layer has been intact (yellow circle). (d) The peroral cholangioscopy examination showed a single white‐toned papillary tumor with an irregular surface at the same site

**FIGURE 3:**
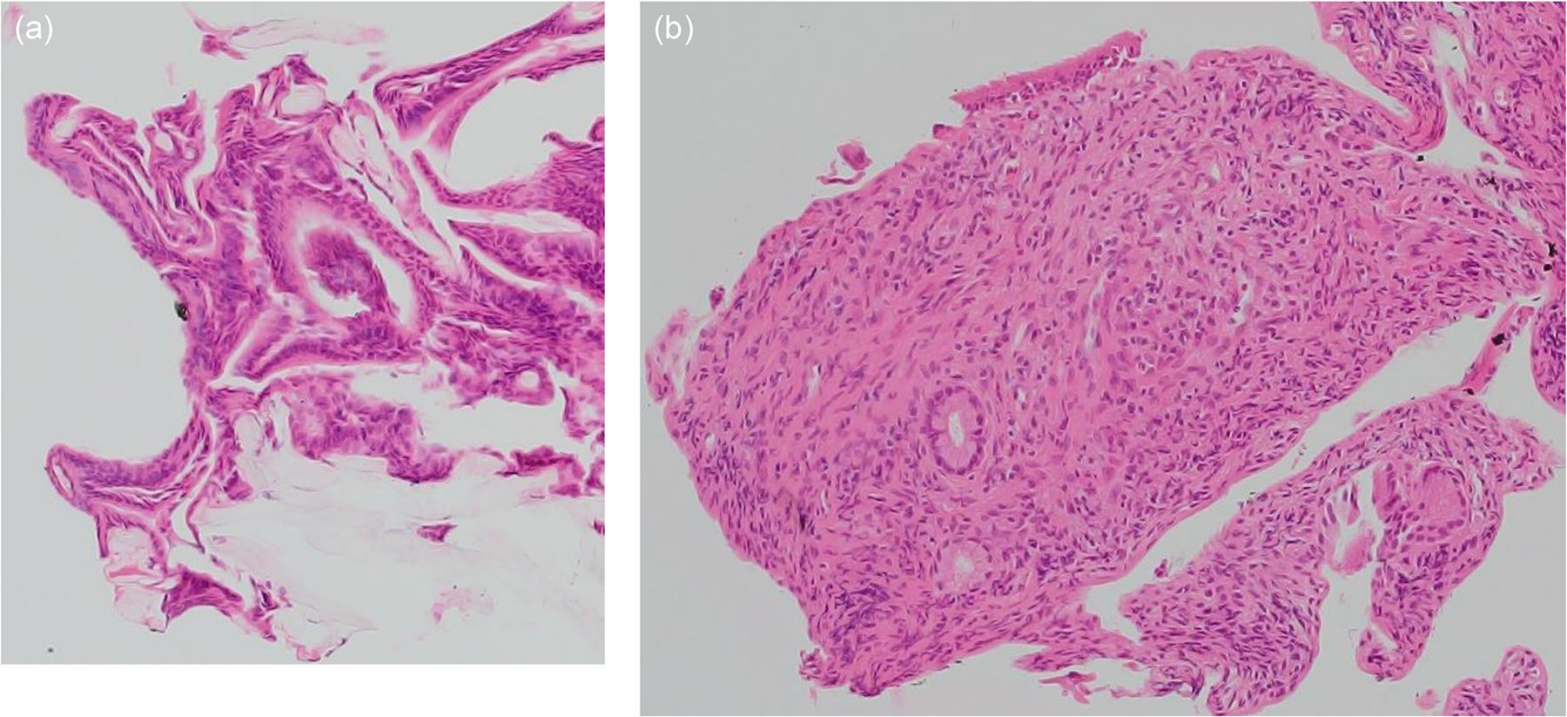
Pathological examination of the bile duct biopsy. Pathological examination of the surface of the tumor biopsy revealed the atypical epithelium in the form of a papilla, and hyperplasia of the glandular epithelium was observed, resembling a gastric type adenoma (a) hematoxylin/eosin (HE, ×100), and the biopsy of the base of the tumor revealed a small number of epithelial and stroma with moderate lymphocyte infiltration (b) (HE, ×100)

**FIGURE 4:**
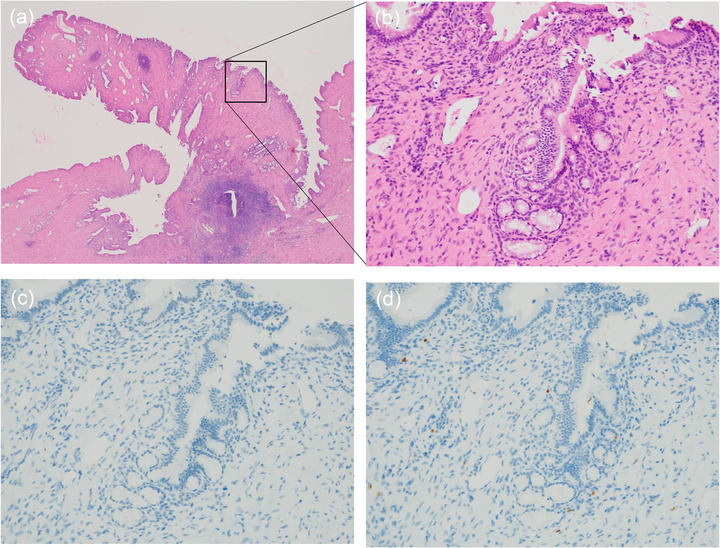
Histopathological examination of the excised surgical specimen. (The tumor was histopathologically identified as a polypoid‐raised lesion with a superficial layer covered with gallbladder‐specific epithelium to the pylorus‐like epithelium and poor atypical epithelium (a) (HE, ×20) and (b) (HE, ×200). Immunostaining did not show proliferation of p53‐positive or Ki67‐positive cells (c) (P53, ×200) and (d) (Ki67, ×200)

A 69‐year‐old woman was hospitalized due to epigastric discomfort. She was diagnosed with cholangitis. There was no increase in white blood cell count at the time of admission, but total bilirubin, aspartate aminotransferase, alanine aminotransferase, lactate dehydrogenase, alkaline phosphatase, and gamma‐glutamyl transpeptidase were 0.8 mg/dl (normal range, 0.4–1.6 mg/dl), 376 IU/L (7–38 IU/L), 370 IU/L (4–44 IU/L), 457 IU/L (120–240 IU/L), 706 U/L (38–113 IU/L), and 234 U/L (<30 IU/L) respectively, indicating liver and biliary tract injury. The serum level of the tumor marker carcinoembryonic antigen and carbohydrate antigen 19‐9 before bile duct drainage had normal values of 0.6 ng/ml (<5.0 ng/ml) and 3.1 U/ml (<37 U/ml), respectively. Wall thickening near the hilar area and the distal bile duct junction was discovered using abdominal contrast‐enhanced computed tomography (CT). There was no evidence of gallbladder edema or intrahepatic bile duct dilation (Figure 1a–c), and magnetic resonance cholangiopancreatography (MRCP) revealed a lack of signal at the transition between the hilar region bile duct and the distal bile duct (Figure 1d). Based on imaging data, she was suspected of having bile duct cancer in the hilar region, and cholangitis was caused by bile duct stenosis. ERCP was conducted with a duodenoscope (TJF‐260V; Olympus, Tokyo, Japan) to cure cholangitis and examine the bile duct stenosis in detail. Small CBD stones were suspected, and an endoscopic sphincterotomy was performed to remove small CBD stones (Figure 2a, yellow arrow). For the lesion suspected of cholangiocarcinoma by the contrast‐enhanced CT and MRCP, the cholangiography findings showed an intraductal mass image near the hilar region of the bile duct (Figure 2b). Intraductal ultrasonography (IDUS) using a Miniature Probe (UM‐DG20‐31R; Olympus) imaging data, which were subsequently done during ERCP, revealed the fact that the bile duct's exterior layer is preserved (Figure 2c). The POCS examination, performed with an oral cholangioscope (CHF‐B290; Olympus), revealed a single white‐toned papillary tumor with an uneven surface at the same location and no abnormally proliferating and tortuous vascular structure (Figure 2d). Then, we took biopsies at two different spots on the tumor: the surface and the base. Pathological examination of the biopsy of the surface of the tumor biopsy revealed atypical epithelium in the form of a papilla, as well as hyperplasia of the glandular epithelium, which resembled a gastric type adenoma (Figure 3a), and the biopsy of the tumor base revealed a small number of epithelial and stroma with moderate lymphocyte infiltration (Figure 3b). Although no clear malignant signs were found, we could not rule out IPNB in this tumor. IDUS observation indicated tumor invasion in the bile duct epithelium warmed the malignant tumor. From some clinical diagnoses, we could not completely deny IPNB or biliary malignant tumor, so we obtained adequate informed permission and decided to conduct extrahepatic bile duct resection to avoid over‐surgery. If the quick intraoperative pathology diagnosis revealed malignant findings, pancreaticoduodenectomy was planned. The extrahepatic bile duct resection went on as planned, with no malignant findings in the quick pathological diagnosis. The tumor was histopathologically identified as a polypoid‐raised lesion with a superficial layer covered with gallbladder‐specific epithelium and poor atypical epithelium (Figure 4a,b). Immunocytochemistry revealed little evidence of p53‐positive or Ki67‐positive cell growth (Figure 4c,d). The presence of malignant epithelial growth was unclear, and the pathological diagnosis was inflammatory bile duct polyp.

## DISCUSSION

The most prevalent causes of CBD obstruction are bile duct stones or malignant strictures; however, inflammatory polyps in the extrahepatic bile duct are a rare disease. There have been few reports of CBD‐related benign tumors, such as adenomas, papillomas, or polyps.^4^ Burhans and Myers reported that papilloma was found in 44 cases and adenoma in 39 of 88 cases of extrahepatic bile duct polyps.^5^ The clinical entity IPNB was first defined in 2010.^2^ IPNB is defined as a general term for tumors that grow in the bile duct, such as a pedunculated papilla, dilate into a spindle or sac, or change to a polycystic cyst.^3^ Almost all previous cases of CBD polyps can now be classified as IPNB, and surgical excision is advised for IPNB without distant metastases since it has high malignant potential and frequently causes recurrent cholangitis and obstructive jaundice. Only 19 cases of inflammatory polyps in the CBD have been reported, according to our search for reports on inflammatory polyps in the CBD. Mechanical stimulation is thought to be one of the causes of inflammatory polyps in CBD. T‐tubes were placed in four cases, biliary stones were discovered in 15 cases, and one of the previous cases had pancreaticobiliary maljunction. Because it is common in situations of bile duct stone problems and T‐tube insertion, it is assumed to be caused by direct physical irritation to the bile duct mucosa or cholangitis.^4^ Although it is an old report, there is a report that denies the role of stones because the number of cases of stones is 78.6% according to the report by Inomata et al. However, there is a report in Europe and the United States that denies the participation of stones because it is little as 8%–12%.^6^ It was assumed in this case that the presence of CBD stones, as well as direct chronic physical irritation to the bile duct mucosa induced by cholangitis, caused bile duct inflammatory polyps. Kim et al. reported that the tumor vessel of the bile duct was defined as an abnormally proliferating and tortuous vascular structure, and by combining this tumor vessel observation with a histopathologic evaluation of percutaneous transhepatic cholangiography‐guided biopsies, the rate of positive diagnosis was significantly increased compared with that made with biopsy or tumor vessel observation alone (*p* < 0.05).^7^ They concluded these tumor vessel finding imaging may be useful for the benign or malignant diagnosis of biliary tumors. In our case, the POCS finding imaging had no tumor vessels as an abnormally proliferating and tortuous vascular structure, so the finding tumor, in this case, was that of a benign tumor according to the results of this paper. Even though we achieved benign results in preoperative biopsy and direct observation, preoperative denial of cancer was difficult in this patient.

## CONFLICT OF INTEREST

The authors declare no conflict of interest.

## FUNDING INFORMATION

None.

## ETHICS STATEMENT

All procedures have been performed in accordance with the ethical standards of the Declaration of Helsinki and its later amendments. The written consent of the patient has been obtained.
